# Accuracy and Precision of Alchemical Relative Free Energy Predictions with and without Replica‐Exchange

**DOI:** 10.1002/adts.201900195

**Published:** 2019-11-27

**Authors:** Shunzhou Wan, Gary Tresadern, Laura Pérez‐Benito, Herman van Vlijmen, Peter V. Coveney

**Affiliations:** ^1^ Centre for Computational Science, Department of Chemistry University College London London WC1H 0AJ UK; ^2^ Computational Science Laboratory Institute for Informatics Faculty of Science University of Amsterdam Amsterdam 1098XH The Netherlands; ^3^ Computational Chemistry, Janssen Research & Development Janssen Pharmaceutica N. V. Turnhoutseweg 30 B‐2340 Beerse Belgium

**Keywords:** binding free energies, FEP+, replica‐exchange, thermodynamic integration with enhanced sampling

## Abstract

A systematic and statistically robust protocol is applied for the evaluation of free energy calculations with and without replica‐exchange. The protocol is based on ensemble averaging to generate accurate assessments of the uncertainties in the predictions. Comparison is made between FEP+ and TIES—free energy perturbation and thermodynamic integration with enhanced sampling—the latter with and without the so‐called “enhanced sampling” based on replica‐exchange protocols. Standard TIES performs best for a reference set of targets and compounds; no benefits accrue from replica‐exchange methods. Evaluation of FEP+ and TIES with REST—replica‐exchange with solute tempering—reveals a systematic and significant underestimation of free energy differences in FEP+, which becomes increasingly large for long duration simulations, is confirmed by extensive analysis of previous publications, and raises a number of questions pertaining to the accuracy of the predictions with the REST technique not hitherto discussed.

## Graphical abstract

A comparison between the TIES and FEP+ alchemical free energy methods for calculating relative free energies of binding of ligands to a target protein is reported. TIES is found to perform best. Replica‐exchange methods exhibit artefactual reduction in these free energy differences, the FEP+ predictions degrading increasingly with longer simulation time.

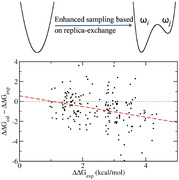

## 1 Introduction

Notwithstanding numerous false dawns, we are now approaching an era when rapid, reliable, and reproducible free energy predictions for ligand–protein binding are becoming available.^1^ Advances in free energy calculations have been fostered by the integration of improved force fields, enhanced sampling methods and increased computer power. The quality of predictions is understood to be determined by two principal sources: one is of a systematic nature owing to the force fields selected, the system setup, the algorithms, and their implementation, the sampling methodology, the extent of configurational space sampling, and so on;^2^ the other is associated with the intrinsic stochasticity of the molecular dynamics simulation method used to compute binding affinities.^3^ The accuracy is dominated by the former, while the precision is mainly determined by the latter.

Protein force fields are now generally quite reliable, although ligand parameterization for new molecules can still pose issues for modelers. The system setup needs to capture essential chemical details and cannot be overlooked; the challenge to model ionization and tautomeric states accurately in a molecular mechanics formalism is a limitation (this is distinct from actual force field errors).1a The extent of conformational sampling also determines the quality of the calculations, as it is not possible to sample the entire conformational space for a complex molecular system, the most relevant subspace for a given thermodynamic property needs to be well sampled to obtain a converged ensemble average. It should be noted, however, that an apparently converged average, with no obvious change in time after a given period, does not necessarily mean that the relevant conformational space has been adequately sampled.2b

The stochastic uncertainty in predictions emanating from molecular dynamics simulations originates in the intrinsically chaotic behavior of the trajectories, as these display extreme sensitivity to initial conditions.^3^ Historically, and still to an overwhelming degree today, an assumption is made that a single “long time” average provides the ensemble average from which statistical mechanics delivers macroscopic averages in the thermodynamic limit. However, these “long time” averages show no convincing tendency to converge to ensemble averages, which today can often be determined directly on large supercomputers.^3^, ^4^

Such uncertainty quantification (UQ) furnishes a statistical estimate of the reproducibility of results between theory/simulation and experiment, and from two or more theoretical methods. Making comparisons can never be done from “one off” molecular dynamics results; both experiment and theory are riddled with errors and uncertainties and the issue is how to reliably compare them. This requires not only computational results to be reported with errors, but likewise experiments.^5^

The ensemble approach is, in the more traditional jargon of molecular dynamics, a highly effective way of “sampling.” It is also a standard procedure adopted for UQ.^6^ There have been many other efforts to accelerate sampling as compared to the traditional one of a single MD run. One such method is called replica‐exchange with solute tempering (REST2)^7^ in which interactions of a chosen subsystem with its environment are scaled to enhance overall conformational sampling for the regions of interest. REST2 is a modified version of the original “replica‐exchange with solute tempering” (REST1)^8^: in REST1, the entire solute biomolecule is heated up while in REST2 the hot region is restricted to a local region of the solute. In Schrödinger's proprietary FEP+ package, the REST2 method is implemented but called REST. For the sake of simplicity and consistency, hereafter we will call the approach REST. REST has been implemented in several molecular simulation packages including NAMD,^9^ Amber,^10^ GROMACS^11^ as well as within the FEP+^12^ package. As we shall see, these methods do not necessarily perform more accurately than ensemble averaged calculations of free energies without such “enhanced sampling.” There are claims that REST improves the convergence of free energy computations,^13^ and hence reduces the variations in the prediction. Our previous study,5b however, has shown that the calculated free energy differences from five replicas vary by up to 1.2 kcal mol^−1^ from TIES calculations with REST employed. Established “enhanced sampling” methods also require ensemble averaging as the stochastic variation is intrinsic to all MD‐based methods.^3^, ^5^

Recently at Janssen, FEP+ simulations have been applied to different drug discovery projects,^14^ and at UCL we have studied various series of ligands bound to a given protein target^5^, ^15^ as well as the same ligands to wild‐type and mutant proteins.^16^ We have introduced variants of TIES, which incorporate the “enhanced sampling” REST techniques including the popular free energy estimator MBAR (multistate Bennett acceptance ratio).5b We have also extended the TIES methodology to study relative binding affinities caused by protein mutations when bound to a ligand, a variant which we call TIES‐PM.^16^ With REST, TIES‐PM can capture large conformational changes; for example, it generates correct free energy differences caused by the gatekeeper mutation occurring inside the ligand binding pocket of the FGFR‐1 kinase.^16^ Simulations using standard TIES, without REST, cannot overcome significant energy barriers between conformations and hence the results are highly sensitive to the initial structures. Nonetheless, we have also observed cases where the application of REST degrades the quality of free energy predictions.^16^ These particular cases demand extensive evaluation with large datasets to take them out of the class of the anecdotal and into the domain of the scientific.

The purpose of the present paper is to assess the performance of “sampling” strategies in two approaches to relative free energy prediction, TIES with and without replica‐exchange along with FEP+. As noted above, a recent paper reported a direct comparison between TIES computed using NAMD and pmemdGTI, where the same force field was used but with different protocols.5b Here we use the reference data set from previous publications^12^, ^15^ to look at the predictions from the TIES and FEP+ protocols. The paper is structured as follows: in the next section, we lay out the methods used; in the following one, we present the results and a discussion. The paper ends with our conclusions from the study.

## 2 Experimental Section

The molecular systems and the simulation protocols are summarized in **Table** 1. Three molecular systems were used: BRD4 (bromodomain containing 4),15c MCL1 (myeloid cell leukemia 1), and TYK2 (Tyrosine kinase 2).^12^, ^15^ The same molecular systems as used before with the TIES approach were studied,15a,c allowing comparison of the accuracy of normal TIES with FEP+ and TIES with REST (TIES‐λ‐REST). The same initial structures and same ligand pairs as in previous studies15a,c were used for both FEP+ and TIES‐λ‐REST simulations. The simulation‐ready molecular systems, in Amber format for TIES and maegz format for FEP+, can be found in Supporting Information.

**Table 1:** Molecular systems and simulation protocols

	TIES	TIES‐λ‐REST (‐MBAR)	FEP+
Molecular systems	BRD4 (12 ligand pairs); MCL1 (16 ligand pairs); TYK2 (11 ligand pairs)		
Force field	Amber ff99SBildn		OPLS3e
MD engine	NAMD 2.9	NAMD 2.11^a^	Desmond v3.8.5.19
Topology	Dual topology		Single topology
Box buffer [Å]	14		10
Timestep [fs]	2		4/8
Cut‐off [Å]	12		9
λ windows	13		12
Protocol	5 replicas, 4 ns production run for each λ window		
Extension^b^	–	4 ns, 20 replicas	4 ns, 30 replicas
		40 ns, 10 replicas	40 ns, 20 replicas
Hours/ns^c^	1.41^d^	2.36^e^	0.05^f^

a Customized version of the NAMD 2.11 package with a patch to implement the REST algorithm for alchemical simulations;

b 5 ligand pairs are chosen for each protein system;

c use TYK2 as an example;

d 128 cores on SuperMUC for one λ window;

e 124 cores on BlueWaters for one λ window;

f 4x Nvidia Tesla K80.

### 2.1 TIES‐λ‐REST Simulations

For the purpose of comparison, the same Amber ff99SBildn^17^ force field was used in TIES‐λ‐REST as in TIES calculations.15a,c The same procedures to set up the protein−ligand systems as recently reported and validated were used.5b A customized version of the NAMD 2.11 package,^18^ with implementation of REST for alchemical simulations,^9^ was used for all the TIES‐λ‐REST simulations. A dual topology scheme was employed to describe a hybrid ligand which consisted of both a disappearing and an appearing group. The two groups define all the alchemically mutating atoms, exclusively belonging to the two ligands which are transferred from one to another during the alchemical process. Thermodynamic integration was used to calculate the free energy changes Δ*G*_alch_ for the ligand pairs in protein and in solvent. The binding free energy difference ΔΔ*G*_cal_ was then calculated as the differences of the ΔG_alch_ values from the two simulations. The REST region for unbound ligand calculations was defined as the set of alchemically mutating atoms. For bound ligand calculations, the REST region comprised all alchemically mutating atoms and all protein residues within 3 Å distance of the former. All TIES(‐λ‐REST) simulations use 13 λ windows per perturbation. A soft core potential was applied for the van der Waals interactions of all atoms in the alchemical space. No soft‐core potential was used for the electrostatic interactions. For the disappearing atoms, the electrostatic interactions were linearly decoupled from the simulations between λ values of 0 and 0.55 and completely turned off beyond that; for the appearing atoms, they were linearly coupled to the simulations from λ value 0.45–1, and fully extinguished otherwise. The approach of decoupling/coupling at different rates ensured that the partial charges were removed on perturbed atoms before they were fully annihilated, while the charges on the growing atoms were introduced after they appeared.

Each REST simulation involves running a predefined number of parallel REST replicas, 13 in the TIES‐λ‐REST simulations, varying in both their effective temperatures and the alchemical parameter λ.^16^ Regular exchange of configurations was attempted between neighboring REST replicas. All TIES‐λ‐REST simulations were run on the BlueWaters supercomputer at the National Center for Supercomputing Applications of the University of Illinois at Urbana−Champaign and Titan at Oak Ridge National Laboratory. The previous TIES calculations were run on the SuperMUC Phase 1 and 2 computers at the Leibniz Supercomputing Centre (Table 1). The benchmark simulations showed that TIES‐λ‐REST consumed about 10–20% more node hours than the standard TIES approach.

### 2.2 FEP+ Approach

FEP+ calculations were performed using Maestro v2018.2, Desmond multisim version 3.8.5.19 and mmshare version 4.2, along with the first version of the proprietary OPLS3e force field. A recent OPLS3e paper^19^ showed that some changes had been made since OPLS3, one being replacement of a quantum chemical MP2 calculation with a density functional method for the torsions, another being an extended chemical environment for torsion definition. A REST enhanced sampling technique was used in the Desmond MD engine, with the same effect as REST in TIES‐λ‐REST simulations. The default FEP+ protocol was used to define the REST region in which only perturbed ligand atoms were included for the simulations in water and in protein complexes (note that a different REST region was used in TIES‐λ‐REST for complex simulations). Missing force field parameters were added by additional QM calculations^19^ and fitted using the ffbuilder module. A single topology scheme was used, in which corresponding atoms were mapped between the two ligands via a maximum common substructure search. No cycle‐closure averaging and error estimation were used as the free energy changes were calculated individually for each ligand pair. FEP+ uses 12 λ windows per perturbation in both solvent and complex, and makes use of a mixed coupling/decoupling schedule. Bonded interactions were scaled linearly across all 12 λ windows as they were either removed or introduced. Desmond used soft core potentials to overcome possible van der Waals end point instabilities at the limits of the λ coordinate. For atom deletion, charges were decoupled first in a linear manner during the first five λ windows with the remaining seven λ windows used to turn‐off the van der Waals terms. The reverse schedule was used for introducing atoms. There are other settings in FEP+, which differ from those in TIES simulations; these included a smaller box size, a smaller cut‐off distance for non‐bonded interactions, and larger time steps (Table 1). All FEP+ Desmond runs were performed on an in‐house GPU (Nvidia Tesla K80) cluster at Janssen Research & Development, Beerse, Belgium.

### 2.3 Simulation Protocol

For both TIES‐λ‐REST and FEP+ simulations, the protocol established in the previous publications was used, in which an ensemble of five replicas had been employed.5b,a It should be noted that such ensemble‐based simulation is not part of the “standard” FEP+ protocol. All replicas have identical initial coordinates but different velocities drawn randomly from a Maxwell−Boltzmann distribution. 4 ns production runs were performed for each replica.15a The protocol of five replicas and 4 ns production times had been shown to produce accurate, precise, and reliable relative free energies in the previous TIES studies on various molecular systems,^5^, ^15^, ^16^ of which a subset was invoked to make a direct comparison between approaches with the replica‐exchange method, as well as between its use and non‐use. The MBAR approach was automatically applied in FEP+ simulations to generate free energy estimates. For TIES‐λ‐REST, the free energy differences both with and without MBAR were reported. The protocol of five replicas and 4 ns production runs were established using TIES, with or without REST.^5^, ^15^ To test this, in the unlikely case that a different combination might favor FEP+, the number of replicas and the duration of the production MD runs were also varied. Thus, some simulations were extended up to 30 replicas and 40 ns (Table 1). The accuracy of free energy approaches was therefore evaluated by comparing the calculations with the experimental data using mean signed errors (MSEs), mean unsigned errors (MUEs), root‐mean‐square errors (RMSEs), and linear regression. For the experimental data without uncertainties reported, an approximate error of 0.3 log units (0.41 kcal mol^−1^) was used.^20^ The way TIES computes precisions was from the TI integral correctly interpreted as a stochastic integral.^3^, ^15^ For FEP+, the error was provided for each individual replica according to standard MBAR theory. The MBAR errors, however, largely underestimate the variances of the free energy results from replica calculations (see Section 3). To avoid such issues and make the performance of the TIES‐based approaches and FEP+ comparable, the standard deviations were used here to assess the precision for all of the approaches.

## 3 Results and Discussion

**Figure 1:**
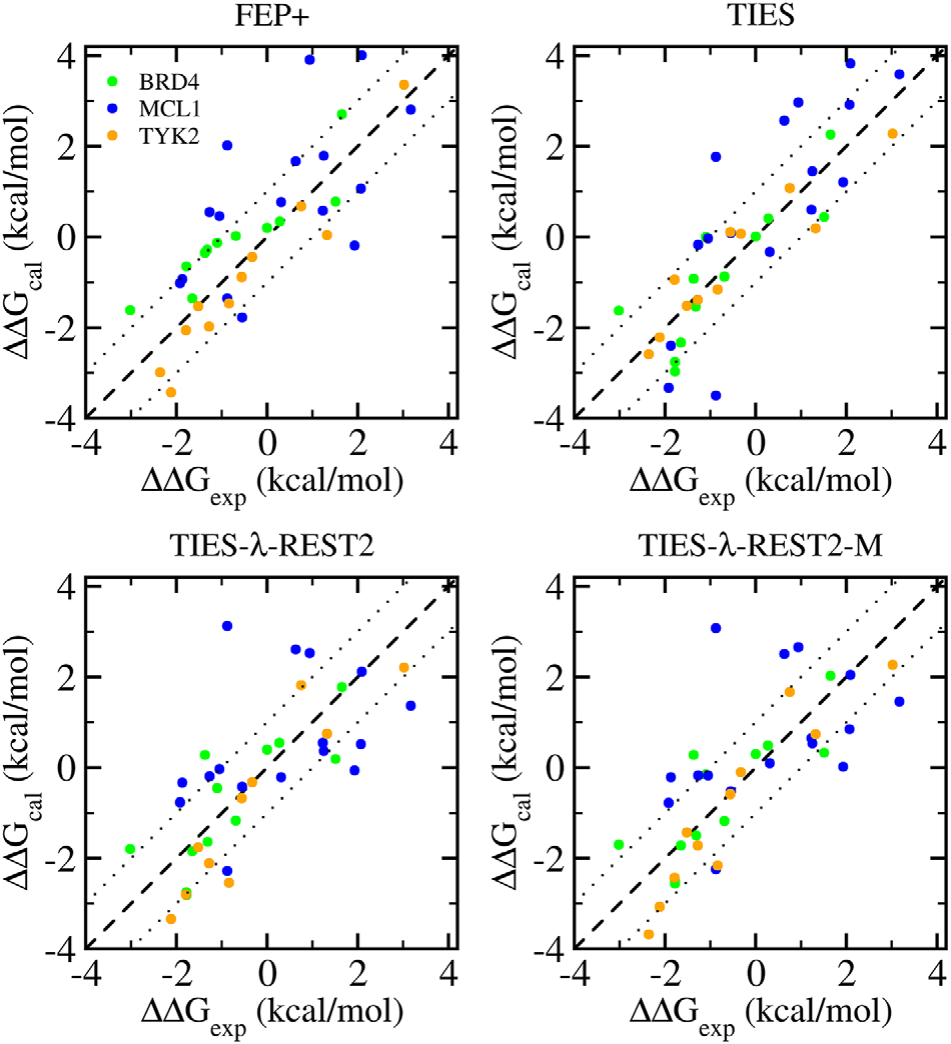
Comparison of the predicted binding free energy differences with the experimental data from the four approaches. See Table 2 for the errors and correlations.

In this section, we compare the accuracy and precision of free energy estimates from different approaches: TIES, TIES‐λ‐REST (‐MBAR), and FEP+. Between TIES‐λ‐REST and TIES‐λ‐REST‐MBAR, we consider the former for reasons of simplification, as the two approaches generate very similar results in terms of MSEs, MUEs, RMSEs, and correlation coefficients (**Table** 2). Our previous studies have shown that MBAR does little to enhance such free energy predictions.^5^, ^16^ All approaches generate good predictions when compared with the experimental data (**Figure** 1 and Table 2). Taking together all of the quantifications in Table 2, it can be seen that TIES yields the best results, with the smallest MSEs, MUEs, and RMSEs and the best correlations in most cases. It should be said that these statistical differences are only marginal. The predictions from FEP+ are comparable to the results from TIES‐λ‐REST.

**Table 2:** Free energy predictions from FEP+, original TIES, TIES‐λ‐REST, and TIES‐λ‐REST‐MBAR. All the results are from ensemble simulations consisting of five replicas and 4 ns production runs each. Standard deviations in parentheses

Protein	Property	FEP+	TIES	TIES‐λ‐REST	TIES‐λ‐REST‐M
BRD4	No. of pert.	12			
	MUE	0.81 (0.14)	0.67 (0.15)	0.72 (0.16)	0.69 (0.15)
	MSE	−0.59 (0.22)	0.00 (0.25)	−0.09 (0.27)	−0.16 (0.25)
	RMSE	0.91 (0.14)	0.81 (0.16)	0.87 (0.17)	0.84 (0.16)
	Pearson r	0.90	0.84	0.80	0.81
	Slope	0.72	0.92	0.82	0.81
	intercept	0.47	−0.11	−0.13	−0.03
MCL1	No. of pert.	16			
	MUE	1.30 (0.24)	1.20 (0.23)	1.34 (0.28)	1.26 (0.25)
	MSE	−0.22 (0.42)	0.27 (0.39)	−0.71 (0.43)	−0.64 (0.39)
	RMSE	1.53 (0.27)	1.41 (0.26)	1.61 (0.33)	1.56 (0.31)
	Pearson r	0.61	0.80	0.41	0.44
	Slope	0.68	1.18	0.37	0.40
	intercept	0.68	0.32	0.44	0.48
TYK2	No. of pert.	11			
	MUE	0.51 (0.16)	0.44 (0.15)	0.85 (0.21)	0.66 (0.17)
	MSE	0.27 (0.26)	−0.25 (0.23)	0.60 (0.32)	0.36 (0.28)
	RMSE	0.67 (0.19)	0.56 (0.17)	1.02 (0.23)	0.79 (0.19)
	Pearson r	0.97	0.94	0.93	0.94
	Slope	1.11	0.83	1.20	1.11
	intercept	−0.39	−0.12	−0.55	−0.38
All	No. of pert.	39			
	MUE	0.93 (0.13)	0.82 (0.12)	1.01 (0.15)	0.91 (0.13)
	MSE	−0.20 (0.20)	0.04 (0.19)	−0.15 (0.23)	−0.21 (0.21)
	RMSE	1.16 (0.16)	1.05 (0.16)	1.26 (0.19)	1.18 (0.18)
	Pearson r	0.78	0.84	0.72	0.74
	Slope	0.87	1.04	0.81	0.79
	intercept	0.29	0.14	−0.13	−0.02

Mean unsigned error $\mathrm{MUE}=\sum_{i=1}^{n}\frac{|\Delta \Delta G_{\exp}-\Delta \Delta G_{\mathrm{cal}}|}{n}$; mean signed error $\mathrm{MSE}=\sum_{i=1}^{n}\frac{\Delta \Delta G_{\mathrm{cal}}-\Delta \Delta G_{\exp}}{n}$ (rearrange each ligand pair so that ∆∆*G*_exp_ ≥ 0); root mean squared error $\mathrm{RMSE}=\sqrt{\frac{1}{n}\sum_{i=1}^{n}{\left(\Delta \Delta G_{\exp}-\Delta \Delta G_{\mathrm{cal}}\right)}^{2}}$. Slope (*m*) and intercept (*b*) are defined as in $\Delta \Delta G_{\mathrm{cal}}=\alpha *\Delta \Delta G_{\exp}+\beta$.

While ensemble approaches diminish random errors from simulations, they cannot remove systematic bias, a deviation of a measurement or prediction from the true value. Indeed, recent work shows that even ensemble averages are likely to contain systematic errors, caused by a newly discovered pathology of floating point numbers.^21^ The possible bias for each of the above approach is indicated by the derivation of the slope from one in the linear regression $\Delta \Delta G_{\mathrm{cal}}=\alpha *\Delta \Delta G_{\exp}+\beta$, and can be quantified by the MSE values (Table 2). An overestimation is defined by a positive MSE, (ΔΔ*G*_cal_ – ΔΔ*G*_exp_) > 0 and a slope greater than one when ligand pairs are rearranged so that ΔΔ*G*_exp_ >_ _0; conversely, an underestimation arises when (ΔΔ*G*_cal_ – ΔΔ*G*_exp_) < 0 and the slope < 1. TIES results exhibit negligible bias with small MSEs for each protein system and for the entire data set. Indeed, TIES generates an average overestimation of 0.27 kcal mol^−1^ for MCL1, an underestimation of 0.25 kcal mol^−1^ for TYK2, no bias (0 kcal mol^−1^) for BRD4, and no bias (0.04 kcal mol^−1^) for the entire dataset; the slope for the fitted line is also close to one for the entire dataset. Conversely, FEP+ manifests a bias in the case of BRD4 with 0.59 kcal mol^−1^ underestimation, while generating equal but opposite MSEs for the other two systems. For the entire data set, FEP+ underestimated the relative free energy changes by 0.20 kcal mol^−1^. TIES‐λ‐REST shows similar bias for each perturbation as FEP+ but with different magnitudes, and a similar underestimation for the entire dataset. It should be noted, however, that the dataset used here is still relatively small; a large and broad dataset with multi‐target and multi chemotype will be required to have more statistically significant conclusions.

**Figure 2:**
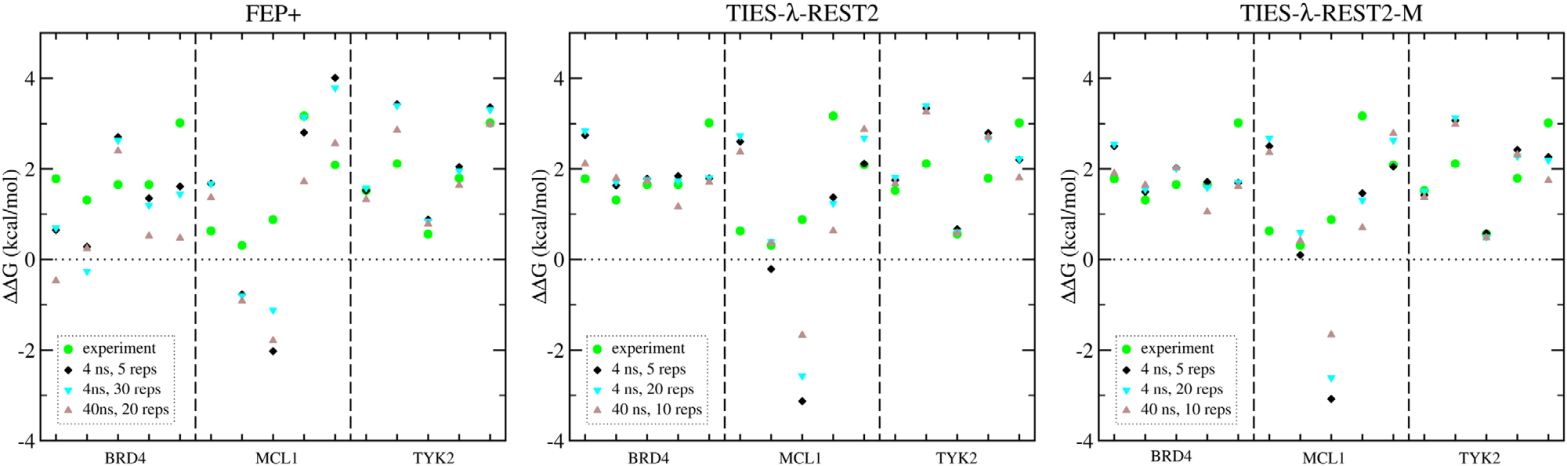
Changes of predicted binding free energies when the number of replicas is increased and/or the length of simulations is extended.

Using more replicas does not confer significant benefit on the predictions of binding free energy differences in either FEP+ or TIES‐λ‐REST (‐MBAR); MUEs, MSEs, RMSEs, and correlation coefficients are all comparable between the predictions from simulations with five replicas and 20–30 replicas (**Table** 3). Likewise, for predictions using standard TIES5b and the TIES‐λ‐REST approach, longer simulation time does not make a significant impact on the predictions, based on the values of MUEs, MSEs, RMSEs, and correlation coefficients (Table 3 and **Figure** 2, confirming our earlier findings). However, and by contrast, in the FEP+ approach longer simulation time degrades performance, which is discussed further below. It renders the underestimation even more severe, as indicated by the MSE values that increase from 0.13 to 0.65 kcal mol^−1^ for the subset of ligand pairs for which the simulation duration is increased by an order of magnitude (Table 3).

**Table 3:** Results of FEP+ and TIES‐λ‐REST(‐MBAR) with different numbers of replicas and simulation lengths. Standard deviations in parentheses

Protein	Property	FEP+			TIES‐λ‐REST			TIES‐λ‐REST‐MBAR		
		4 ns 5 reps	4 ns 30 reps	40 ns 20 reps	4 ns 5 reps	4 ns 20 reps	40 ns 10 reps	4 ns 5 reps	4 ns 20 reps	40 ns 10 reps
BRD4 MCL1 TYK2	No. of pert.	15								
	MUE	0.96 (0.21)	0.91 (0.18)	1.04 (0.22)	0.97 (0.28)	0.96 (0.27)	0.92 (0.23)	0.86 (0.28)	0.89 (0.26)	0.89 (0.23)
	MSE	−0.13 (0.35)	−0.13 (0.32)	−0.65 (0.33)	−0.15 (0.40)	−0.03 (0.40)	−0.16 (0.36)	−0.22 (0.38)	−0.12 (0.38)	−0.25 (0.24)
	RMSE	1.21 (0.26)	1.10 (0.21)	1.33 (0.25)	1.40 (0.39)	1.32 (0.35)	1.23 (0.27)	1.33 (0.39)	1.28 (0.36)	1.19 (0.27)
	Correlation	0.87	0.89	0.78	0.77	0.79	0.79	0.78	0.80	0.80
	Slope	1.01	1.00	0.69	0.87	0.91	0.81	0.85	0.87	0.77
	intercept	0.52	0.52	−0.48	−0.02	0.03	−0.03	0.12	0.17	0.08

**Figure 3:**
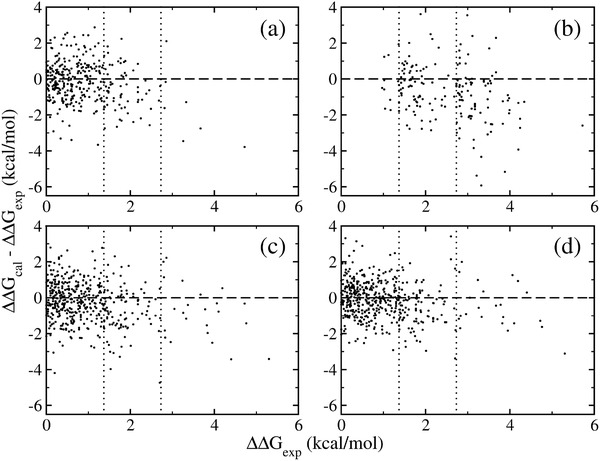
Underestimation of the relative free energy differences. Data are taken from previous FEP+ calculations from a) Wang et al.,^12^ b) Pérez‐Benito et al.,^20^ c) Roos et al.^19^ using OPLS3, and d) Roos et al.^19^ using OPLS3e force field. See Table 4 for quantitative assessments.

The underestimation of FEP+ relative free energy calculations has been recently noted.^20^ To further quantify the underestimation in FEP+ calculations, we revisit three sets of predictions from two Schrödinger publications^12^, ^19^ and one set from the recent study by Pérez‐Benito et al.^20^ (see **Figure** 3 and **Table** 4). These studies all looked at relatively large datasets and thus produce reliable statistics. The original ΔΔ*G*_FEP+_ values were obtained from Supporting Information^12^, ^19^ and from the authors.^20^ In the original FEP+ paper with OPLS2.1 force field,^12^ a 0.18 kcal mol^−1^ underestimation is manifest for the entire dataset consisting of 330 alchemical mutations. Using the latest versions of the OPLS force field, similar underestimations have been observed.^19^ These underestimations are comparable with the MSE value (0.20 kcal mol^−1^, Table 2) in the current study. The underestimation in FEP+ is much more obvious and severe when the binding free energy differences are large. When the ΔΔ*G*_exp_ values are in the range of 1.37–2.73 kcal mol^−1^, 1–2 log units in activity, the underestimation is ≈0.50 kcal mol^−1^; when the difference is more than 2 log units (2.73 kcal mol^−1^), the underestimation can be as much as 1.56 kcal mol^−1^ (Table 4). The comparison of the results from 1 and 5 ns simulations^20^ shows that long simulations degrade the quality of FEP+ predictions; longer simulations make them even worse, as observed in the current study (Table 3).

**Table 4:** Revisit binding free energy differences (kcal mol^−1^) of FEP+ calculations in literature

	|ΔΔ*G*_exp_| < 1.37	1.37 ≤ |ΔΔ*G*_exp_| < 2.73	|ΔΔ*G*_exp_| ≥ 2.73	Total
Wang et al.,^12^ OPLS2.1, Figure 3a				
No. of transformations	257	66	7	330
Underestimations [%]	51	71	86	55
(ΔΔ*G*_cal_–ΔΔ*G*_exp_)/*N*	−0.04	−0.59	−1.56	−0.18
Roos et al.,^19^ OPLS3, Figure 3c				
No. of transformations	348	98	23	469
Underestimations [%]	55	71	74	59
(ΔΔ*G*_cal_–ΔΔ*G*_exp_)/*N*	−0.12	−0.71	−0.82	−0.28
Roos et al.,^19^ OPLS3e, Figure 3d				
No. of transformations	348	98	23	469
Underestimations [%]	52	71	65	57
(ΔΔ*G*_cal_–ΔΔ*G*_exp_)/*N*	−0.03	−0.53	−0.51	−0.16
Pérez‐Benito et al.,^20^ all ligand pairs with 5 ns simulations from LO datasets, Figure 3b				
No. of transformations	10	79	92	181
Underestimations [%]	70	65	83	74
(ΔΔ*G*_cal_–ΔΔ*G*_exp_)/*N*	−0.13	−0.43	−1.14	−0.78

“Underestimations” is the percentage of pairs of which ΔΔ*G*_cal_ < ΔΔ*G*_exp_ when the ligand pairs are rearranged so that ΔΔ*G*_exp_ ≥ 0 kcal mol^−1^. The average underestimation (ΔΔ*G*_cal_ – ΔΔ*G*_exp_)/*N* is also calculated after making the ΔΔ*G*_exp_ ≥ 0 rearrangement.

REST has been implemented in FEP+ and TIES‐λ‐REST approaches. A larger database will be required to confirm the apparent underestimations of the free energy differences in TIES‐λ‐REST‐MBAR. Although the quality of these simulation results may be affected by many factors,2b we suspect that the REST protocol is the dominant reason for the underestimations exhibited in these simulations. TIES and TIES‐λ‐REST(‐MBAR) simulations share the same protocol including the force field and the initial structures. The only difference is the use of REST in TIES‐λ‐REST (‐MBAR), which is likely to be the reason that TIES calculations outperform TIES‐λ‐REST (‐MBAR) in its relative free energy predictions. The REST approach enhances conformational sampling, but by the same token is able to reach conformations which are less relevant to stable binding and can produce diminished differences in binding affinities for pairs of congeneric ligands, as recently reported.^16^ The lack of the correct weighting of these less relevant states in REST‐implemented calculations causes an artefactual reduction in the difference of the binding free energies.^16^ Longer REST simulations increase the occurrence of such conformations and hence make the predictions increasingly unreliable. For the FEP+ calculations, the force field may also contribute to the bias, as the latest OPLS3e force field reduces the underestimations in the free energy predictions as compared with OPLS3 force field (Table 2 and ref. [19]).

**Figure 4:**
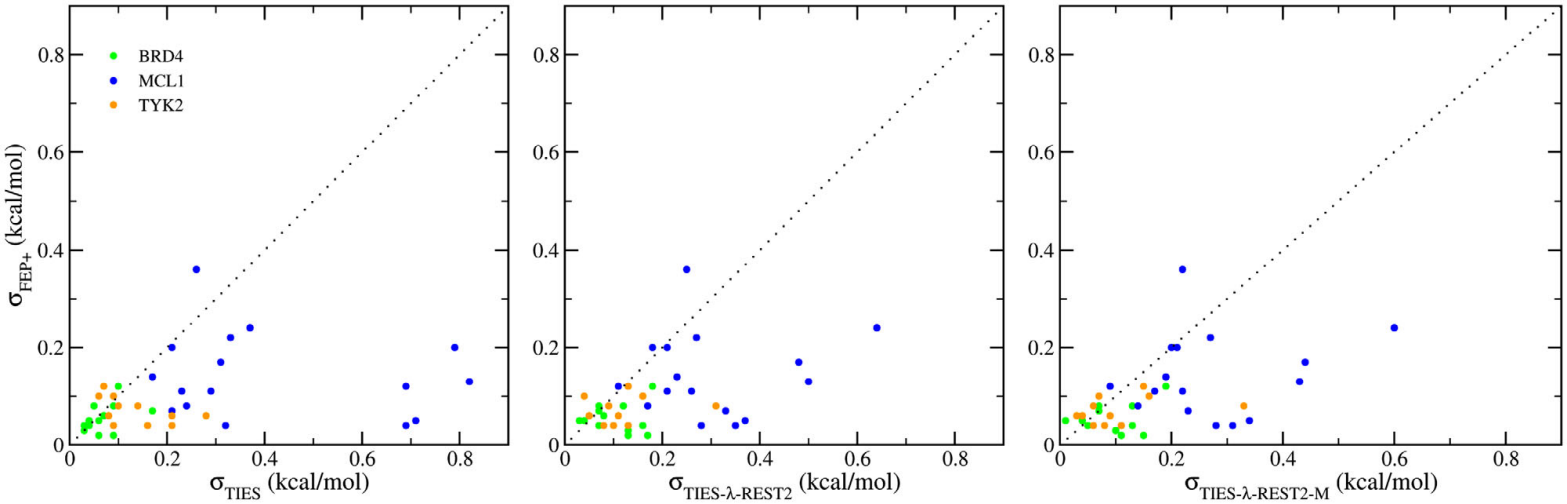
Comparison of the bootstrapping errors of FEP+ with TIES, TIES‐λ‐REST, and TIES‐λ‐REST‐MBAR.

Although FEP+ manifests smaller variations between different replicas than TIES or TIES‐λ‐REST(‐MBAR) (**Figure** 4), it produces much larger variations from 30 replicas than the MBAR errors reported for each FEP+ calculation (**Table** 5). The results from single FEP+ calculations can vary by up to 3.94 kcal mol^−1^ for one ligand pair to MCL1. Although the implementation of an accelerated sampling protocol such as REST may reduce the variations from independent runs, the results here show again that one‐off runs are not reliable. Statistical properties derived from ensembles are much more robust.

**Table 5:** Variations and ranges of binding free energy differences (kcal mol^−1^), compared with the averaged MBAR errors, from 30 replicas of 4‐ns FEP+ calculations. Standard deviations in parentheses

Protein	Ligand pair	Average	Range from 30 replicas	*σ*_MBAR_
BRD4	02–03	−2.64 (0.15)	−3.02 to −2.38 (0.64)	0.08
	03–06	−1.20 (0.27)	−1.80 to −0.69 (1.11)	0.12
	08–09	1.44 (0.16)	0.98 to 1.71 (0.73)	0.09
	13–14	−0.70 (0.30)	−1.35 to −0.12 (1.23)	0.11
	14–15	0.26 (0.10)	0.14 to 0.47 (0.33)	0.04
MCL1	02–32	−1.11 (0.95)	−3.44 to 0.50 (3.94)	0.31
	08–18	−3.79 (0.23)	−4.21 to −3.09 (1.12)	0.07
	16–34	0.80 (0.11)	0.53 to 1.06 (0.53)	0.05
	32–38	−3.15 (0.54)	−4.30 to −2.09 (2.21)	0.19
	35–12	1.67 (0.34)	0.95 to 2.27 (1.32)	0.07
TYK2	01–03	0.85 (0.28)	0.36 to 1.58 (1.22)	0.12
	01–08	1.96 (0.17)	1.49 to 2.22 (0.73)	0.10
	01–10	3.40 (0.23)	2.90 to 3.89 (0.99)	0.11
	06–11	−3.31 (0.17)	−3.79 to −2.90 (0.89)	0.08
	06–15	−1.58 (0.17)	−1.94 to −1.21 (0.73)	0.08

It is likely that the number of atoms in the alchemical region plays an important role in accounting for the differences of errors from TIES and FEP+ (Figure 4). FEP+ implements a single topology approach which morphs as many atoms as possible that are different between the two end states. TIES and its variants use a dual topology approach in which the appearing and disappearing groups are not morphed and move independently. If we define dummy atoms as those having no interactions with the environment at one or the other end state, the number of dummy atoms is usually significantly larger in a dual topology than in a single topology scheme. These dummy atoms can sample different conformational spaces and may be very flexible, especially when their interactions with the environment are scaled down. This introduces larger variations in the energies and energy derivatives in TIES and TIES‐λ‐REST(‐MBAR) than FEP+.

## 4 Conclusion

In this study, we compare the accuracy and precision of relative free energies calculated from standard TIES15a,c and two REST‐implemented approaches: TIES‐λ‐REST (with or without MBAR)5b and FEP+.^12^ The performance of standard TIES has been compared with pmemdGTI in our previous study.5b For the reference data set investigated, standard TIES performs best. The protocol of a 4 ns production run and five replicas, as established in our previous TIES studies,^5^, ^15^, ^16^ is reasonable for TIES and its variants, since neither an increase in the number of replicas nor the duration of simulations have a large impact on the predictions, as adjudged by the quantities MUEs, MSEs, RMSEs, and correlation coefficients (Figure 2 and Table 3). Indeed, an interesting conclusion from this study is that more replicas does not confer significant benefit on the predictions of binding free energy differences. However, FEP+ accuracy deteriorates as the simulation duration is extended.

The REST‐implemented calculations all show an underestimation of computed relative free energies, which are especially noticeable when the experimental binding free energy difference is large. Longer simulations degrade predictions in FEP+ when conformations are sampled which are less relevant to stable ligand binding. Proper weighting is required for the entire conformational space sampled to produce reliable free energy predictions. However, it is difficult to evaluate the likelihood of a conformation and hence its contribution to the predictions.^16^ Force fields can also contribute to the observed bias in relative free energy predictions; the latest OPLS3e force field improves the FEP+ results but does not remove its systematic underestimations.

## Conflict of Interest

The authors declare no conflict of interest.

## Supporting information

Supporting Information

Supporting Information
